# Overexpression of CAPN2 promotes cell metastasis and proliferation via AKT/mTOR signaling in renal cell carcinoma

**DOI:** 10.18632/oncotarget.22083

**Published:** 2017-10-26

**Authors:** Chenkui Miao, Chao Liang, Ye Tian, Aiming Xu, Jundong Zhu, Kai Zhao, Jianzhong Zhang, Yibo Hua, Shouyong Liu, Huiyu Dong, Chao Zhang, Shifeng Su, Pu Li, Chao Qin, Zengjun Wang

**Affiliations:** ^1^ State Key Laboratory of Reproductive Medicine and Department of Urology, The First Affiliated Hospital of Nanjing Medical University, Nanjing, China; ^2^ Department of Urology, Nanjing First Hospital, Nanjing Medical University, Nanjing, China

**Keywords:** CAPN2, renal cell carcinoma, metastasis/proliferation, EMT, AKT/mTOR signaling

## Abstract

The calpain 2 (CAPN2) is upregulated in various malignant carcinomas. Previous studies have reported that CAPN2 functioned as an oncogenic factor in human cancers. However, its clinical role and potential effects on cell metastasis and proliferation in renal cell carcinoma (RCC) remain unknown. In this study, we evaluated the mRNA and protein levels of CAPN2 in human RCC specimens, matched normal specimens, and RCC cell lines using quantitative Real-time PCR (RT-PCR) and western blot. Immunohistochemistry of 74 RCC tissues in a tissue microarrays (TMAs) and normal kidney tissues were performed. Kaplan-Meier survival curve analyses were conducted to measure the correlation between CAPN2 and tumor prognosis. Cell migration, invasion and proliferation were detected by transwell assays and Cell Counting Kit-8 (CCK-8) assays. CAPN2 exhibited a significant overexpression in human RCC tissues and cell lines compared with adjacent non-tumor tissues and normal human proximal tubule epithelial cell line HK-2. Strong staining of CAPN2 was associated with higher clinical stage and histological grade. In addition, sh-CAPN2 could significantly inhibit migration, invasion and proliferation of 769-P and CAKI-1 cells. Conversely, increased cell biological behaviors were observed in CAPN2-OV CAKI-2 cells. Moreover, the subsequent mechanism investigation suggested that CAPN2 promoted tumor progression by activating AKT/mTOR signaling, enhancing epithelial mesenchymal transition (EMT) and MMP9 levels. The present study indicates that CAPN2 may act as a prominent indicator for RCC progression and a novel therapeutic target for RCC patients.

## INTRODUCTION

Renal cell carcinoma (RCC) is one of the commonest malignant tumors and comprises approximately 90% of kidney cancers [1]. Although RCC represents only 2%–3% of all cancers, a continuing rise in incidence has been observed with 63990 new estimated and 14400 death cases in the United States in 2017 [2, 3]. Operative treatment can cure early-stage RCC, but still almost 30% of patients present with metastatic RCC at the time of diagnosis and 20%–40% develop recurrence after resection [4–6]. It is generally known that RCC is poorly responsive to conventional radiotherapy and chemotherapy contributed by multiple resistance. Therefore, molecular targeting agents such as tyrosine kinase inhibitors, antiangiogenic agents and immunotherapy have become the preferred standard approaches in RCC patients [7–9]. Unfortunately, numerous problems including substantial toxicities, risk of adverse events, and unforeseen efficacy-loss have emerged during the application period, which might attribute to multiple sophisticated mechanisms [10, 11]. Multiple studies have detected numerous biomarkers which involved into cancer progression [12]. Taken together, it is of great necessity to determine novel biomarkers and develop advanced therapeutic strategies for RCC patients.

Calpains family belongs to a family of calcium-dependent protease, of which two ubiquitous isoforms μ-calpain and m-calpain have been well investigated [13]. Calpain 2 (CAPN2), also known as m-calpain, is a member of calpains system. CAPN2 is composed of two subunits: a large 80-kDa catalytic subunit and small 28-kDa regulatory subunit, which can help maintain its biological activity [14]. CAPN2 functions are mainly mediated by Ca^2+^ autoproteolysis, phosphorylation, intracellular distribution and inhibition by calpastatin, among which activation of Ca^2+^ is extensively confirmed [13, 15]. Accumulating experimental and clinical evidence have reported CAPN2 was aberrantly expressed in various tumors and exerted a pivotal role in tumorigenesis and progression. Downregulation of CAPN2 significantly suppressed migration and invasion ability of hepatocellular carcinoma, and attenuated MMP-2 and MMP-9 secretion [16]. In addition, abnormal CAPN2 expression was associated with patients progression-free survival (PFS) and overall survival (OS) in ovarian cancer, and served as a specific prognostic indicator [17]. Furthermore, abnormal CAPN2 level was also detected in metastatic prostate cancer compared with normal tissues, and promoted prostate cells invasive and proliferative capabilities [18–20]. These results highlighted the predictive significance of CAPN2 in metastasis and progression of multiple cancers.

However, little is defined about the biological function of CAPN2 in RCC. In order to identify the functional role of CAPN2, we carried out silencing and overexpressing RCC cell lines to investigate its tumorigenic properties in RCC metastasis, and provided a better potential target for tumor therapy.

## RESULTS

### CAPN2 expression is associated with worse clinical features of RCC

**Figure 1:**
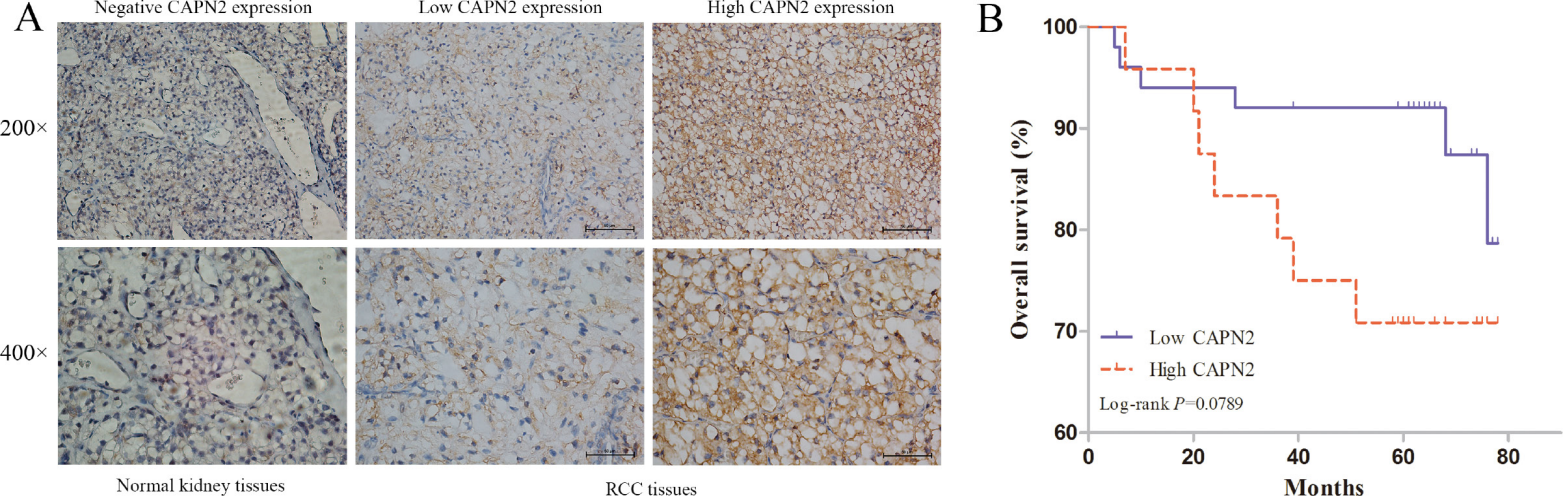
CAPN2 expression in normal kidney tissues and RCC TMAs, and its significance in prognosis of patients (**A**) Immunohistochemistry of CAPN2 protein in normal kidney tissues and RCC TMAs. Negative, low and high CAPN2 expression of immunohistochemical staining were shown with 200×/400×magnification respectively. (**B**) Kaplan-Meier/log-rank analysis exerted no significance between CAPN2 expression and overall survival of RCC patients (*P* = 0.0789).

To validate the clinical value of CAPN2, we determined the protein level of CAPN2 in RCC tissue microarrays of 74 cases and normal kidney tissues by IHC staining. Table 1 summarized the characteristics of RCC patients involved in this study. As shown in Figure 1A, CAPN2 was negatively expressed in normal kidney samples and primarily stained in cytoplasm of tumor cells. Relative expression of CAPN2 of RCC specimens was divided into two groups: high CAPN2 expression (*n* = 50, staining scores ≥2) and low CAPN2 expression (*n* = 24, staining scores <2). Furthermore, results demonstrated that aberrant high CAPN2 level was significantly correlated with advanced tumor stage (*P* = 0.011) and histological grade (*P* = 0.048). However, such meaningful connection was not observed between CAPN2 expression and OS of RCC patients (*P* = 0.102). Association of CAPN2 expression and clinicopathological parameters of RCC was presented detailedly in Table 2. These results strongly suggested that CAPN2 might act as a crucial biomarker for RCC progression.

**Table 1:** Summary of the 74 RCC patients involved in this study

Age		
Mean ± SD, year	56.22 ± 13.32	
<60	42	56.8%
≥60	32	43.2%
Gender		
Male	47	63.5%
Female	27	39.5%
Tumor size		
≤4	42	56.8%
>4	32	43.2%
Histology		
Clear cell carcinoma	61	82.4%
Others	13	17.6%
Histological grade		
I	15	20.3%
II	47	63.5%
III	9	12.2%
IV	3	4.0%
Tumor stage		
T1	64	86.5%
T2	6	8.1%
T3	4	5.4%
Survival		
Mean ± SD, month	60.73 ± 18.27	
No	13	17.6%
Yes	61	82.4%
CAPN2 expression		
Low	50	67.6%
High	24	32.4%

SD, standard deviation.

**Table 2:** Relationship of CAPN2 expression and clinicopathological characteristics of patients

Variable	Total (%)	CAPN2 expression		
		Low (*n* = 50)	High (*n* = 24)	*P* value
Age (year)				0.217
<60	42	31 (73.8)	11 (26.2)	
≥60	32	19 (59.4)	13 (40.6)	
Gender				0.201
Male	47	29 (61.7)	18 (38.3)	
Female	27	21 (77.8)	6 (22.2)	
Tumor size (cm)				0.217
≤4	42	31 (73.8)	11 (26.2)	
>4	32	19 (59.4)	13 (40.6)	
Histology				1
Clear cell carcinoma	61	41 (67.2)	20 (32.8)	
Others	13	9 (69.2)	4 (30.8)	
Histological grade				0.048^*^
I–II	62	45 (72.6)	17 (27.4)	
III–IV	12	5 (41.7)	7 (58.3)	
Tumor stage				0.011^*^
T1	64	47 (73.4)	17 (26.6)	
T2–T4	10	3 (30.0)	7 (70.0)	
Survival				0.102
No	13	6 (46.2)	7 (53.8)	
Yes	61	44 (72.1)	17 (27.9)	

^*^P < 0.05. *P* values were two-tailed and based on the Pearson chi-square test.

### CAPN2 is expressed in RCC tissues and cell lines

**Figure 2:**
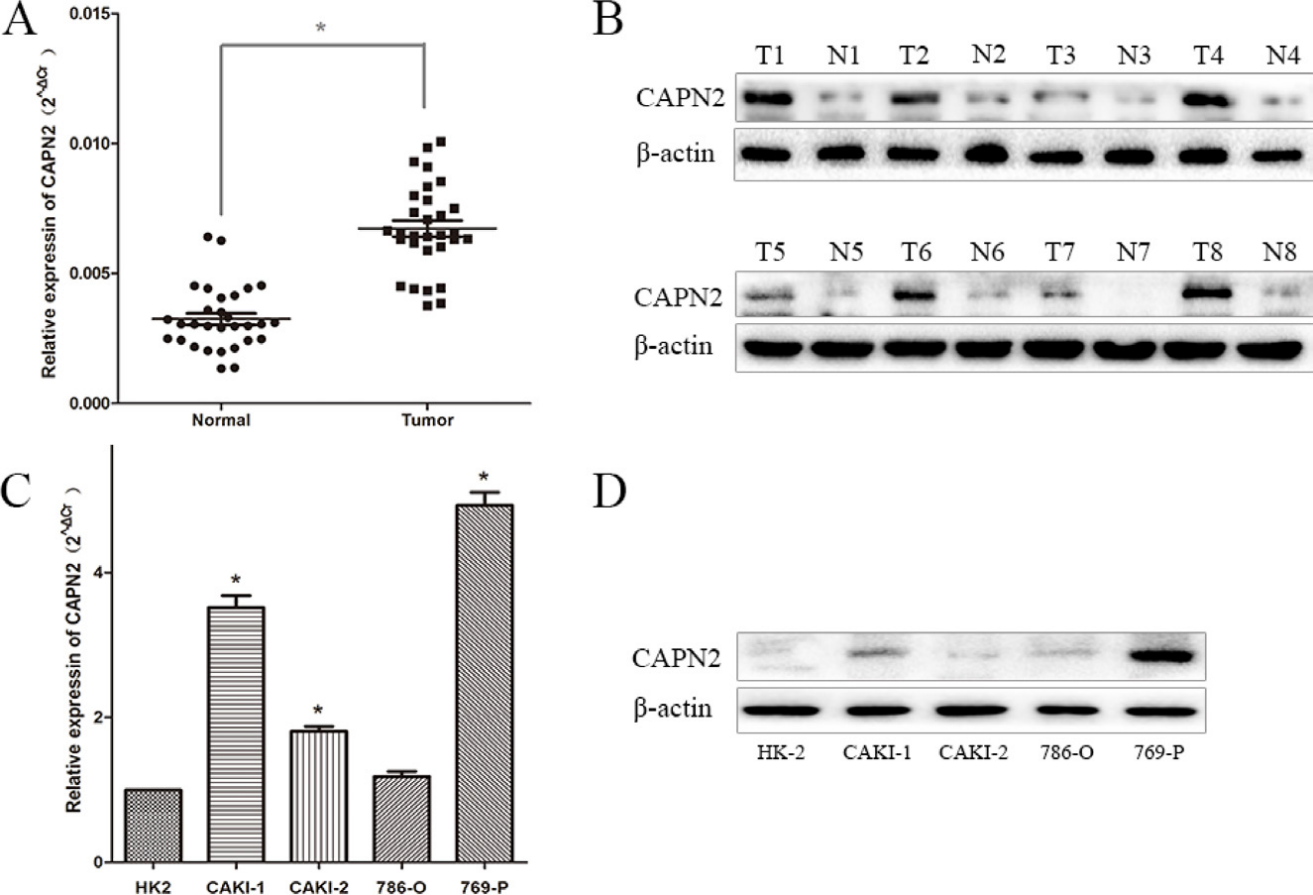
CAPN2 expression in RCC cell lines and tissues (**A**) CAPN2 mRNA expression in 30 pairs of RCC tissues and matched adjacent non-tumorous tissues. (**B**) CAPN2 protein expression in 8 pairs of RCC tissues and adjacent tissues. (**C**) and (**D**) The mRNA and protein expression of CAPN2 in RCC cell lines and normal epithelium cell of renal tubule HK2. Data are the mean ± SD from three independent experiments, ^*^P < 0.05.

**Figure 3:**
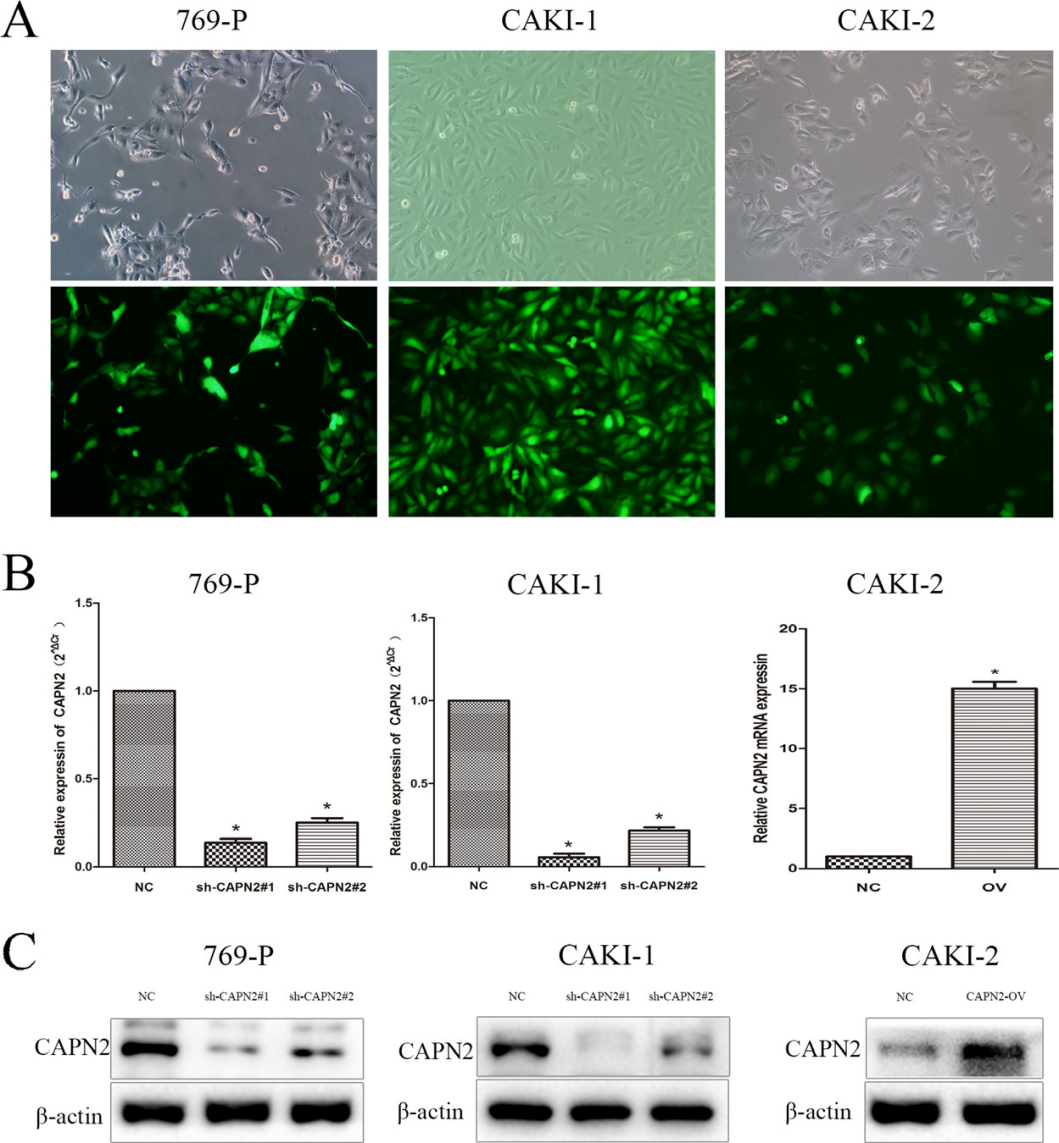
Transfection efficiency of lentivirus in 769-P, CAKI-1 and CAKI-2 cells (**A**) Intracellular fluorescence intensity of GFP was observed under fluorescent microscopy after lentiviral transfection. (**B**) The mRNA expression of CAPN2 after transfection with lentiviral. (**C**) The protein level of CAPN2 after transfection with lentiviral. β-actin was used as an internal control. All data are presented as mean ± SD, ^*^P < 0.05.

To explore the expression level of CAPN2 in RCC cells, we performed quantitative RT-PCR and western blotting in RCC cell lines (CAKI-1, CAKI-2, 786-O, 769-P) and renal tubular epithelial cells HK-2. The mRNA and protein level of CAPN2 were significantly upregulated in 769-P and CAKI-1 compared to HK-2, while results in 786-O cells were not particularly evident (Figure 2C and 2D). In addition, CAPN2 was certainly high expressed in CAKI-2 in mRNA levels but not seriously significant in protein level by western blot assay, which might attribute to the incomplete consistence between mRNA and protein levels. Moreover, the mRNA expression of CAPN2 in RCC tissues were also detected for further understanding. Results demonstrated that CAPN2 mRNA level was markedly evaluated in RCC tissues compared to matched non-tumorous tissues (Figure 2A and 2B). In addition, to further confirm the biological function of CAPN2 in RCC cells, CAPN2 level was blocked down in 769-P and CAKI-1 cells and overexpressed in CAKI-2 cells by transfecting lentivirus vectors (Figure 3A). The silenced cells were named as sh-CAPN2 and overexpressed cells were called CAPN2-OV. Besides, transfection efficiencies of the three cell lines were confirmed by quantitative RT-PCR and western blotting, respectively (Figure 3B and 3C).

### CAPN2 promotes cell migration, invasion and proliferative capability of RCC

**Figure 4:**
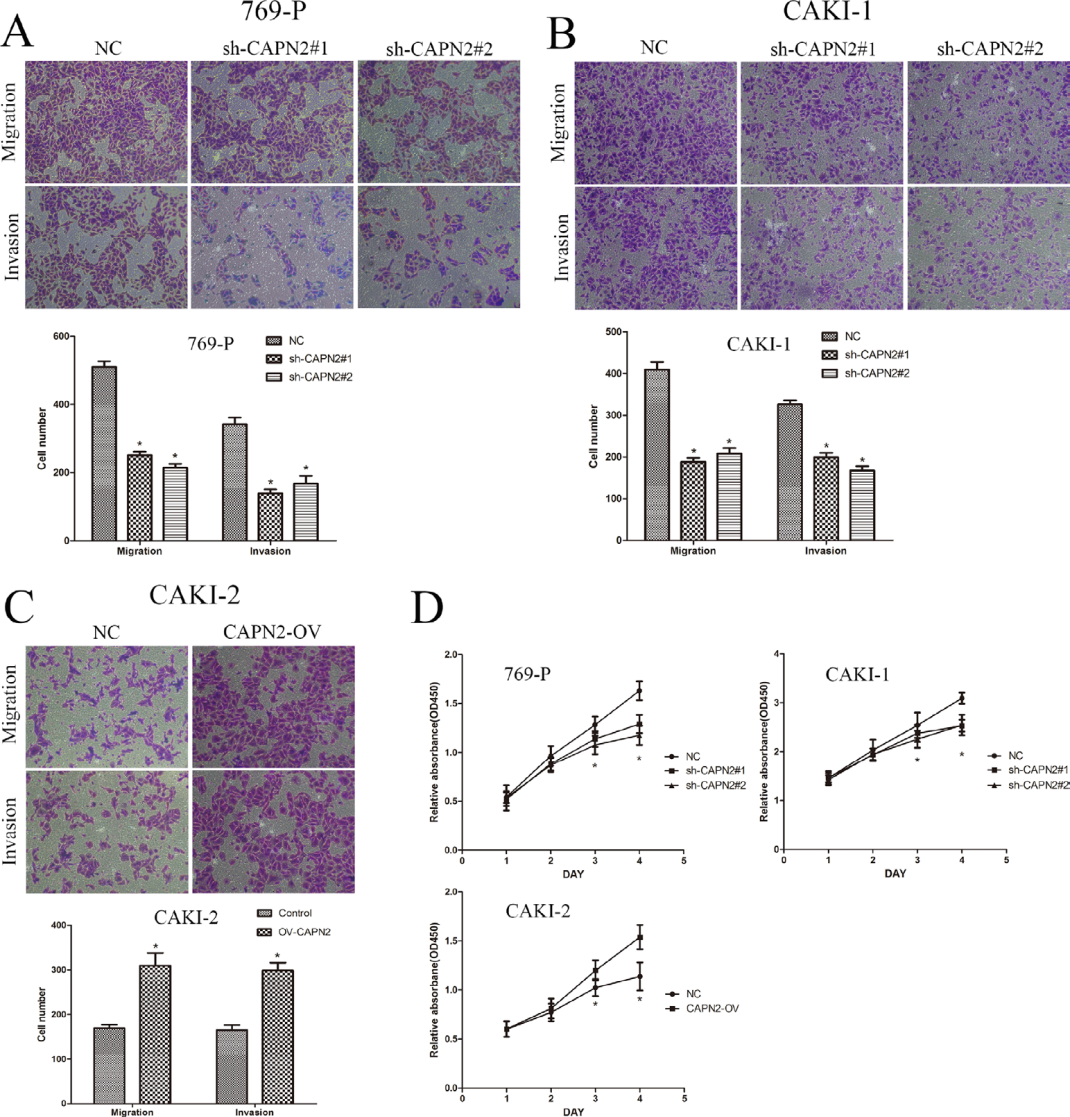
CAPN2 promotes cell migratory, invasive and proliferative potential of RCC cells (**A**–**C**) Transwell migration assay and Matrigel invasion assay in 769-P and CAKI-1 cells with CAPN2 knockdown, in CAKI-2 cells with overexpression of CAPN2. (**D**) Growth curve analysis by CCK-8 assay showed the cell growth of 769-P, CAKI-1and CAKI-2 cells with CAPN2 knockdown or upregulation. Data represented the mean ± SD from three independent experiments, ^*^P < 0.05.

To evaluate the biological potential of CAPN2 in renal cancer, transwell assays were applied to investigate its ability involving in tumor migration and invasion. Lentivirus vectors were transfected to silence CAPN2 expression in 769-P and CAKI-1 cells, and overexpress its level in CAKI-2 cells. As shown in Figure 4A and 4B, sh-CAPN2 significantly decreased the cell numbers of migration and invasion, as measured by transwell assays. Conversely, CAPN2-OV enhanced cell migratory and invasive potential compared with the negative control (Figure 4C). To further confirm the promoting effects of CAPN2 on cell proliferation, we conducted CCK-8 assays in CAPN2 silencing or overexpressing cell lines. Sh-CAPN2 obviously restrained cell proliferation in 769-P and CAKI-1 cells (Figure 4D). Moreover, CAPN2-OV of CAKI-2 cells markedly enhanced cell growth ability, as contrasted with NC group (Figure 4D).

### CAPN2 increases the level of EMT-related markers and MMP9

**Figure 5:**
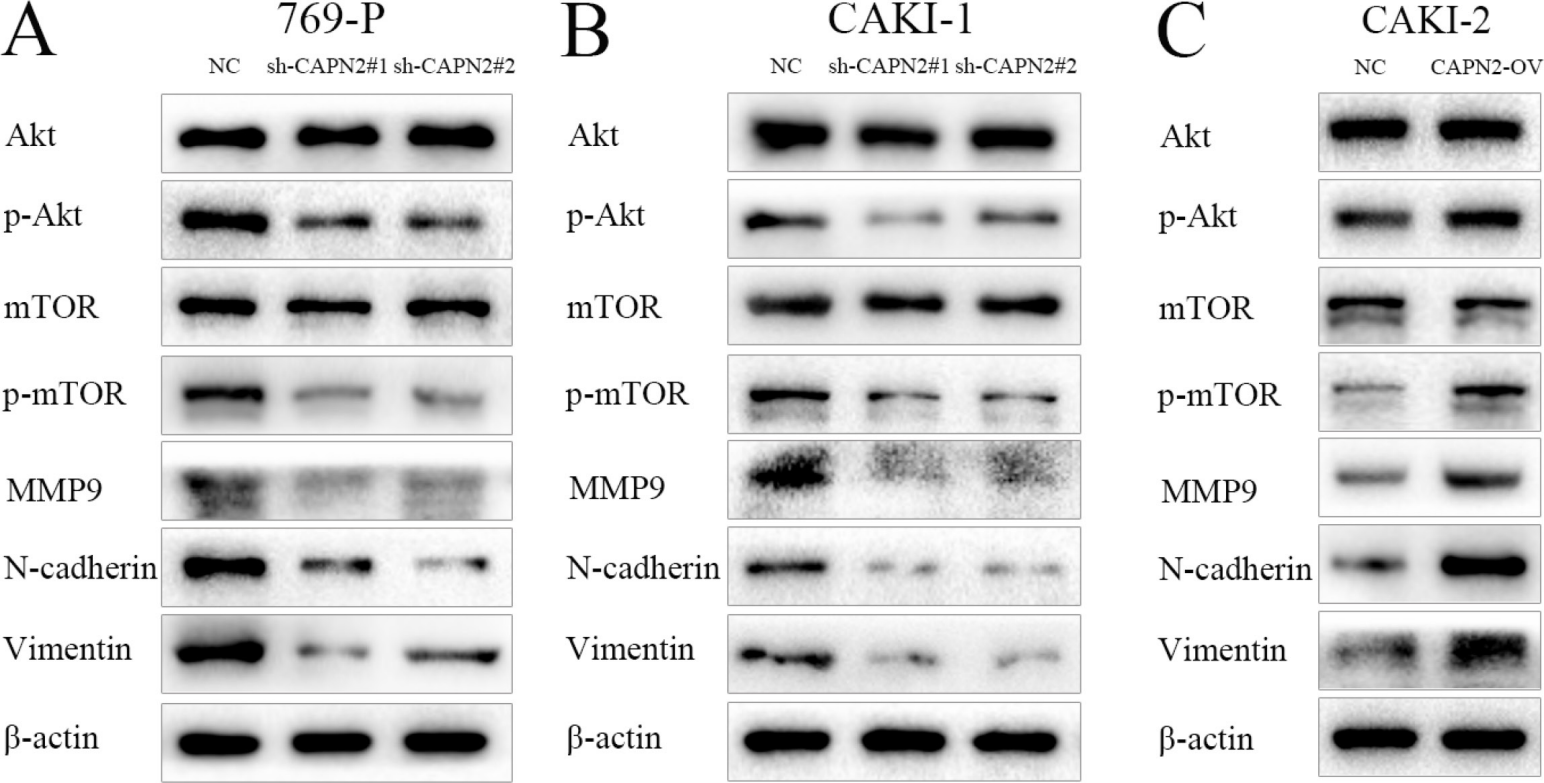
CAPN2 activates AKT/mTOR signaling, and enhances EMT process and MMP9 expression level (**A**–**C**) AKT, phospho-AKT, mTOR, phospho-mTOR protein levels were analyzed by western blot with CAPN2 downregulation or overexpression. MMP9, N-cadherin, and Vimentin protein expression were examined with CAPN2 knockdown or upregulation. Data represent the mean ± SD from three independent experiments, ^*^P < 0.05.

As previously reported, activation of EMT process and MMPs family have been verified to play a substantial role in tumor cell adhesion and metastasis. To further elucidate the underlying mechanisms by which CAPN2 promotes cell migration and invasion, we performed western blot assay to identify the protein level of N-cadherin, Vimentin and MMP9. Compared to NC group, sh-CAPN2 could remarkably inhibit the expression of N-cadherin, Vimentin and MMP9 in 769-P and CAKI-1 cell lines (Figure 5A and 5B). In addition, CAPN2-OV could enhance N-cadherin, Vimentin and MMP9 protein level in CAKI-2 cells, which highlights the promoting role of CAPN2 in tumor metastasis in RCC (Figure 5C).

### CAPN2 promoting action is largely dependent on activation of AKT/mTOR signaling

We next sought to explore the molecule mechanisms by which CAPN2 expression might regulate the proliferation capability of RCC cells. To this end, we investigated the effects of CAPN2 downregulation or upregulation on AKT/mTOR signaling pathway. Results from western blot assays revealed that decreased CAPN2 expression could obviously restrain the level of phospho-AKT and phospho-mTOR in 769-P and CAKI-1 cells, while increased CAPN2 level of CAKI-2 cells activated phospho-AKT and phospho-mTOR expression (Figure 5A–5C). Meanwhile, the total expression levels of AKT and mTOR exhibited no significant discrepancy between sh-CAPN2 and CAPN2-OV RCC cells (*P* > 0.05; Figure 5A–5C). These data indicated that the phosphorylation activation of AKT/mTOR signaling might be involved in the potential mechanisms by which CAPN2 governed RCC progression.

**Figure 6:**
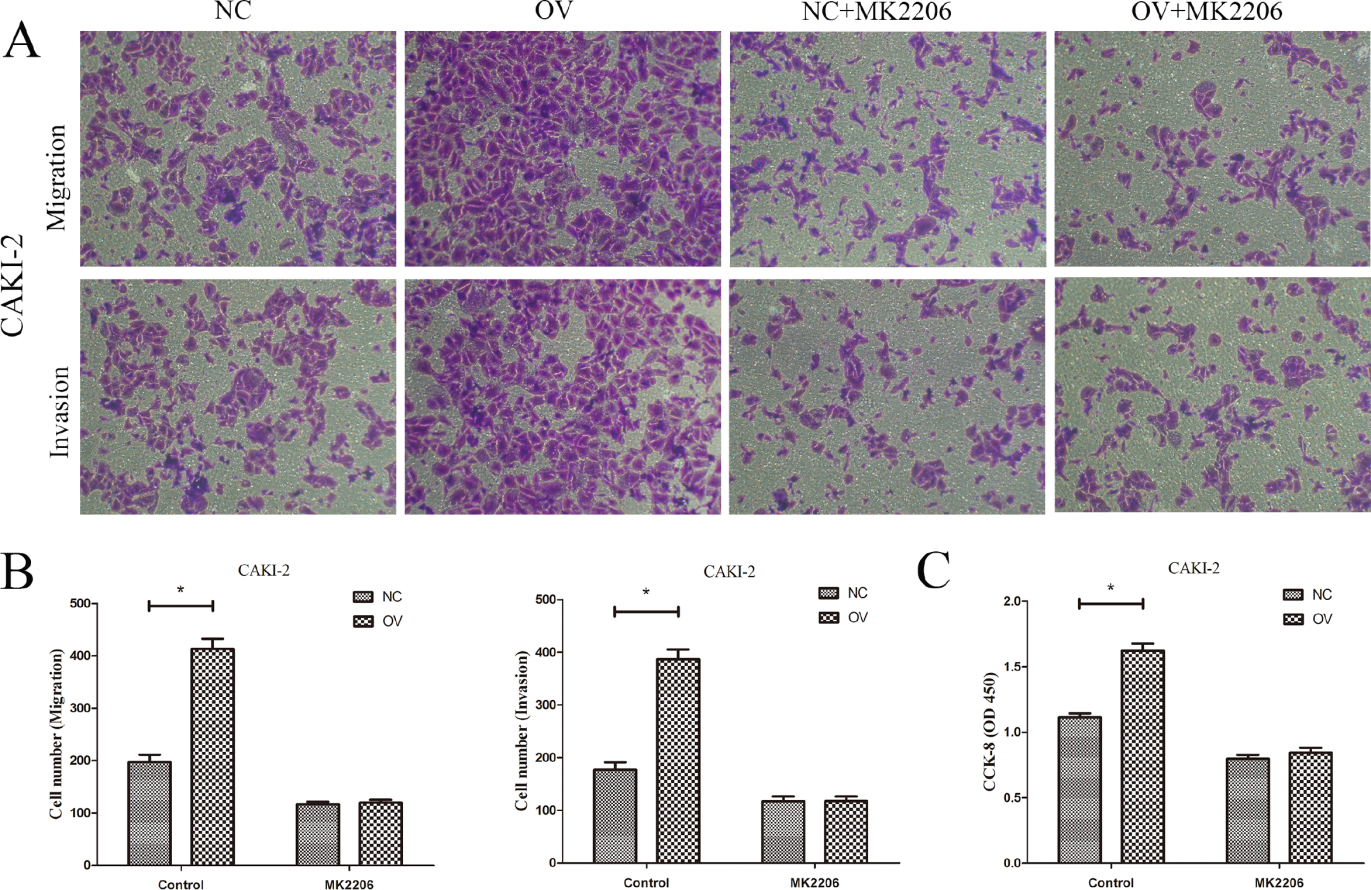
AKT inhibitor MK2206 restrains the oncogenic role of CAPN2-OV in RCC cells (**A**) and (**B**) The promoting role of CAPN2-OV on migration and invasion of CAKI-2 cells was attenuated after adding MK2206. (**C**) The promoting role of CAPN2-OV on proliferative potential of CAKI-2 cells was inhibited by MK2206. Data are mean ± SD of at least three independent experiments, ^*^P < 0.05.

To further validate the mechanism, AKT inhibitor MK2206 (10 ug/ml) was utilized to observe the role of AKT/mTOR signaling in CAPN2-medidated cell metastasis and proliferation. After treated with MK2206 for 24 h, CAKI-2 cells with CAPN2-NC and CAPN2-OV were applied for transwell and CCK-8 assays. It was indicated that CAPN2-OV enhanced cell migration, invasion and proliferation, while MK2206 restrained its promoting role in CAPN2-OV CAKI-2 cells (Figure 6A–6C). These results provided more evidence for the carcinogenic role of CAPN2 in RCC tumor metastasis and proliferation via AKT/mTOR signaling pathway.

## DISCUSSION

With a high metastatic and mortality rate, RCC represents one of the most common difficult-to-cure malignancies, and it has turned to the second leading cause of death among urologic neoplasms [8, 9]. Approximately 30% of patients are diagnosed with metastatic RCC, and 50% of the remaining invalids ultimately develop progression and lead to death [6]. Massive clinical issue has emerged for RCC due to its lacking accurate diagnostic strategies for early stage tumors and predictive biomarkers for progression. Therefore, it is of great urgency to identify novel representative biomarkers for RCC tumor promotion and therapeutic treatment. Accumulating evidence has indicated that CAPN2, a calcium-activated protease, could participate in tumor progression and carcinogenesis in multiple cancer types including prostate cancer, ovarian cancer, breast cancer and hepatocellular carcinoma [16, 17, 20–22]. However, the function of CAPN2 in RCC and its underlying mechanisms remain unclear. Thus, for the first time, we focused on the functional value of CAPN2 and its clinical significance in RCC progression.

In the present study, we demonstrated that CAPN2 expression was markedly evaluated in RCC tissues compared with adjacent non-cancerous tissues. Further analysis of RCC TMAs revealed that high CAPN2 staining was correlated with advanced tumor stage and high histological grade. However, no prominently association was observed between CAPN2 expression and prognostic outcomes in RCC patients. In addition, we also identified CAPN2 was frequently upregulated in RCC cell lines (769-P, CAKI-1, and CAKI-2) compared to normal HK-2 cells, while ACHN and 786-O cells exhibited no obvious difference. To further confirm its potential role in RCC cells, two independent sh-RNAs were utilized to downregulate CAPN2 expression in 769-P and CAKI-1 cells, and lentiviral vector was transfected to overexpress CAPN2 in CAKI-2 cells. Our data suggested that sh-CAPN2 dramatically restrained cell migration and invasion, while CAPN2-OV enhanced migrative and invasive potential. Similarly, sh-CAPN2 also significantly inhibited cell proliferative ability, while upregulation of CAPN2 exerted a promoting function consistently. To conclude, our finding initially suggested that CAPN2 might serve as a novel biomarker and potential target for predicting RCC progression.

Generally speaking, tumor cells migration and invasion are terribly required for cancer metastasis, among which EMT process play a crucial role. EMT, also as epithelial-mesenchymal transition, has been verified to induce the loss of polarity and adhesion features of cells and to gain the mesenchymal phenotype for invasive potential [23–25]. Emerging studies have indicated that EMT stood for a critical step for RCC metastasis and progression [26–28]. Furthermore, tumor progression was also correlated with the reduction of extracellular matrix (ECM) by matrix metalloproteinases (MMPs). MMPs belong to a family of zinc-dependent endopeptidases which have been reported to exhibit marked effects on invasion and metastasis of various cancer types [29–31]. In order to understand the mechanism by which CAPN2 govern RCC metastasis, we detected the expression of EMT-related markers including N-cadherin, Vimentin, and MMP9 protein level in RCC cell lines. CAPN2 could critically increase the expression of N-cadherin, Vimentin and MMP9 proteins, highlighting the molecular mechanism of EMT and MMPs in CAPN2-regulated RCC cells. Therefore, we propose that CAPN2 may promote RCC metastasis and development by mediating EMT process and ECM degradation.

Moreover, mounting evidence has indicated AKT and its downstream molecule mTOR are involved into tumor progression by regulating MMPs family and EMT related markers [32–35]. It has been reported that AKT/mTOR signaling is activated by several tyrosine kinase receptors and exhibit great role in metabolism, proliferation, and protein synthesis in diversified tumor types [36–38]. In a prostate cancer study, abnormal CAPN2 expression was associated with cell metastasis and proliferation by activating AKT/mTOR signaling [20]. Therefore, we further determined the effects of CAPN2 on AKT/mTOR signaling in RCC. Our data suggested that CAPN2 observably enhanced the phosphorylation level of AKT and mTOR, but did not affect the total AKT protein, as well as mTOR. Additionally, AKT inhibitor MK2206 significantly restrained the promoting role of CAPN2-overexpression in RCC cells, which raised the reliability of AKT/mTOR pathway.

To summarize, our study showed the elevated CAPN2 expression in RCC tissues and cell lines, and we first elucidated its biological function in RCC. Overexpressed CAPN2 was involved in advanced tumor stage and histological grade for RCC patients, and played a crucial role in accelerating cell metastasis and proliferation. Hence, our results indicate that CAPN2 may emerge as a specific biomarker and independent predictor for RCC progression. CAPN2 suppression is expected to become a potential target for RCC therapy in the future. Nevertheless, the extensive molecular mechanisms by which CAPN2 mediates the progression of RCC needs further elucidation.

## MATERIALS AND METHODS

### Patients and clinical samples

Thirty pairs of RCC samples and their neighboring tissues were obtained from the patients treated by partial or radical nephrectomy with appropriate informed consent at the First Affiliated Hospital of Nanjing Medical University. The “adjacent normal tissue” was obtained as a 0.5 cm × 0.5cm × 0.5 cm tissue-block far away from the tumor edge at least 3 centimeters; the adjacent normal tissues were obtained as far as possible from the tissues collected from partial or radical nephrectomy. And these samples were immediately frozen in liquid nitrogen and then stored at −80°C for further analysis. The specimens were evaluated by immunohistochemistry and the diagnosis was verified by histopathological examination.

A total of 74 RCC tissues from TMAs were obtained from patients operated by partial or radical nephrectomy at the Department of Urology of the First Affiliated Hospital of Nanjing Medical University from 2008 to 2011. All these patients gave informed consent for their participation in the research. Our study was approved by the Institutional Research Ethics committee of the First Affiliated Hospital of Nanjing Medical University. The follow-up deadline was April 2016. The TMAs included the comprehensive clinical information of the 74 patients and their pathological characteristics were gathered.

### Immunohistochemistry

Consecutive sections derived from TMAs blocks were deparaffinized 2–3 times in xylene for 10 min each and hydrated through an ethanol gradient for 2 min each, and then were blocked in hydrogen peroxide in methanol for 10 min. After a 2-min incubation in a steam pressure cooker containing citrate buffer (10 mM, pH 6.0), the samples complete antigen recovery. Then, the samples were blocked for 5 min and incubated with primary antibodies against CAPN2 (Abcam, UK; 1:100) at 4°C overnight. In the following day, washed with phosphate-buffered saline (PBS), slides were cultured in the secondary antibody at room temperature for 30 min. Having washed in PBS for 10 min, the antibody reaction was visualized in fresh diaminobenzidine (DAB) solution. The sections were counterstained with haematoxylin followed by dehydrating and mounting.

### Evaluation of staining

Staining intensity was scored manually by two independent experienced pathologists without knowledge of the clinical data. In order to assess the expression of CAPN2 in RCC tissues, a semi-quantitative scoring system (0–3) based on the staining intensity of the tumor tissue was used: negative = 0, weak positive = 1, moderate positive = 2, and strong positive = 3. CAPN2 low expression refers to scores 0–1, while CAPN2 high expression refers to scores 2–3.

### Cell culture and transfection

CAKI-1, CAKI-2 and 769-P were obtained from the Cell Bank of Shanghai Institute of Cell Biology (Chinese Academy of Medical Science, Shanghai, China) the Chinese Academy of Sciences (Shanghai, China). The cells were propagated in McCoy's 5A (Gibco, USA) or RPMI-1640 (Gibco, USA) medium supplemented with 10% fetal bovine serum (Gibco, USA) and 1% penicillin/streptomycin (Invitrogen) at an incubator with 5% CO_2_ and 37°C. AKT inhibitor MK2206 (S1078) was obtained from Selleckchem (Houston, Tx, USA).

The lentiviral vectors containing small hairpin RNA (shRNA) targeting CAPN2, overexpression of CAPN2, and negative control (NC) were obtained from Novobio (Shanghai, China). Two shRNAs were called sh-CAPN2#1 and sh-CAPN2#2. The sequence information was listed as the following: sh-CAPN2#1, Top Strand 5′-CACCGGAGCTGCTCTTTGTGCATTCCGAAGAATGCACAAAGAGCAGCTCC -3′, Bottom Strand 5′-AAAAGGAGCTGCTCTTTGTGCATTCTTCGGAATGCACAAAGAGCAGCTCC-3′); sh-CAPN2#2, Top Strand 5′-CACCG*CAGGAACTACCCGAACACATTCGAAAATGTGTTCGGGTAGTTCCTG-3′, Bottom Strand 5′-AAAACAGGAACTACCCGAACACATTTTCGAATGTGTTCGGGTAGTTCCTGC*-3′. Lentiviral transduction was performed in 769-P, Caki-1, and Caki-2 cell lines. Pools of stable transductants were generated by selection using blasticidin (4 μg/ml) for 2 weeks.

### RNA extraction, RT-PCR and quantitative RT-PCR

According to the manufacturer's guideline, total RNA was drawn from cell lines and tissues by using TRIzol reagent (Invitrogen, Carlsbad, CA, USA). Complementary DNA (cDNA) was synthesized by using PrimeScript RT Master Mix (TaKaRa, Kyoto, Japan). Reverse transcription polymerase chain reaction (RT-PCR) was conducted by using GoTaq Green Master Mix (Promega, Fitchburg, WI, USA) in line with the manufacturer's instructions. The PCR products were analyzed on 1% agarose gel and visualized. Quantitative RT-PCR was performed using FastStart Universal SYBR Green Master (Roche, Branford, CT, USA). β-actin was employed as a referencegene to normalize the expression of CAPN2 mRNA. For PCR, we used the following primers (Realgene, Nanjing, China): CAPN2 (5′-AAGTA ACGGAAGCCTACAGAAAC-3′, forward; 5′-ATCTTC ATGCCGTCTGGTCAG-3′, reverse); β-actin (5′-ACTG GAACGGTGAAGGTGAC-3′, forward; 5′-AGAGAAGT GGGGTGGCTTTT-3′, reverse).

### Transwell cell migration and invasion assay

We carried out the cell invasion assay by using transwell chambers of pore size 8 μm (Corning, Inc, Corning, NY, USA) in a 24-well plate. The transfected cells (2 × 10^4^) suspended with serum-free medium were seeded into the upper chamber, while the corresponding medium containing 20% FBS was added into lower chamber. The cells were then allowed to invade the Matrigel matrix for 24 h. The cells were then allowed to invade the Matrigel matrix for 24 h. In the invasion assays, the membranes were coated with Matrigel (BD Biosciences, San Diego, CA, USA) to form matrix barriers. Incubated at 37°C for 48 h (migration assay) or 72 h (invasion assay), the cells on the upper surface were removed with a cotton swab. Thereafter, the cells invaded on the bottom membranes were fixed with paraformaldehyde and stained with 0.5% crystal violet solution. The transmigrated cells were counted in five randomly selected fields under a microscope. All of the experiments were performed in triplicate.

### Cell proliferation assay

A Cell Counting Kit-8 assay (Dojindo laboratories, Kumamoto, Japan) was used to estimate the proliferation potential. Different pretreated cells (2000/well) were seeded in 96-well plates and cultured for 1, 2, 3 and 4 days. CCK-8 reagents were added into wells, and the absorbance was measured at 450 nm using a micro-plate reader at 2 h after CCK-8 addition. Three wells were measured for cell viability in each group.

### Protein isolation and western blot

The transfected cells were lysed in protein lysis buffer (Beyotime Institute of Biotechnology, Nantong, Jiangsu, China) on ice for 30 min. The supernatants were collected after centrifugation for 15 min at 14,000 r/min and the concentration of the protein was calculated by the BCA Protein Quantification Kit (Beyotime Institute of Biotechnology). Proteins were separated by 10% SDS-PAGE gel and transferred onto a polyvinylidene difluoride membrane (Millipore, Billerica, MA, USA) in transfer buffer at 300 mA for 2 h. Then the membranes were incubated with Tris-buffered saline (TBS) containing 5% non-fat milk powder for 2 h at 4°C. The membranes were washed with TBS containing 0.2% Tween- 20 (TBST) three times, a nd then incubated with primary antibodies at 4°C overnight. Later, the membranes were washed with TBST three times and the specimens were hatched with secondary antibody in a secondary antibody solution for 2 h at room temperature. The indicated proteins were detected using a horseradish peroxidase chemiluminescent kit (Thermo Fisher Scientific) using an optional CCD camera and image processing system (Bio-Rad, Hercules, CA, USA). β-actin was used as a loading control. Moreover, N-cadherin, Vimentin, MMP9, AKT, phospho-AKT, mTOR, phospho-mTOR were bought from Cell Signaling Technology, Danvers, MA, USA. The secondary antibodies were also from Cell Signaling Technology.

### Statistical analyses

Data were expressed as mean ± standard deviation (SD) and the statistical calculations were performed with SPSS software (Version 13.0). The chi-squared test was employed to explore the associations between protein expression values and clinicopathological factors. We used the Kaplan–Meier method and log-rank test to assess the association of CAPN2 expression with overall survival. Student's *t*-test and ANOVA were carried out to analyze continuous variables. Differences between two or three groups were compared with Student’ s *t*-test. The value of *P* < 0.05 or less was considered to indicate statistical significance.
